# A Parametric Finite Element Analysis of Chick Embryo Aortic Valve Leaflet Biomechanics

**DOI:** 10.3390/bioengineering13020189

**Published:** 2026-02-06

**Authors:** Onur Mutlu, Sandra Rugonyi

**Affiliations:** Biomedical Engineering Department, Oregon Health & Science University, Portland, OR 97239, USA; mutlu@ohsu.edu

**Keywords:** aortic valve, finite element analysis (FEA), parametric valve modeling, belly curve, chick embryo, leaflet kinematics

## Abstract

The anatomy and mechanical strength of aortic valve leaflets are critical determinants of their biomechanical behavior and long-term structural integrity. The embryonic developmental period, when valves are forming, is critical to establish baseline leaflet properties. However, fetal stages of valve development, when valve leaflets are still forming and remodeling, are not well understood. The goal of this study is to investigate the biomechanical stress and deformation modes of developing valve leaflets during systole, and how leaflet biomechanics are affected by anatomy and material properties. To this end, the study employs a parametric approach to model the leaflet anatomy of an HH40 chick embryo, used here as a model of fetal cardiac development. To perform biomechanical analysis, a pressure profile derived from in ovo Doppler ultrasound measurements was applied, and an Ogden hyperelastic material model was employed following a sensitivity analysis. To determine the effect of valve anatomy on leaflet tissue deformation and stresses, we changed the leaflet midline curve (belly curve) from its native curvature to a linear profile and quantified biomechanical responses. Our analysis revealed a strong decrease in average leaflet effective stress as the belly curvature was shifted towards a linear profile. However, this reduction in average stress was at the expense of a biomechanical trade-off. The shift induced a progressive localization of stress concentration at the leaflet tips and commissures, and a distinct bending deformation mode at the tip under peak load. Our findings demonstrate that while the belly curve of the leaflet modulates tissue stress during valve opening, a low-stress anatomy does not align with hemodynamic performance. This work characterizes competing leaflet biomechanical responses (stress reduction versus failure modes) that shape valve leaflet formation, providing fundamental insights into developmental valve biomechanics.

## 1. Introduction

The aortic valve, one of the four main valves of the heart, functions as a semilunar gateway between the left ventricle and the aorta, ensuring unidirectional systemic blood flow by opening during ventricular systole and closing during diastole [1,2,3]. The aortic valve anatomy consists of three crescent-shaped leaflets with a trilaminar microarchitecture (fibrosa, spongiosa, and ventricularis) that exhibits anisotropic mechanical behavior, allowing the leaflets to undergo large deformations during systolic ejection while sustaining large loads during diastolic closure [4,5,6,7,8]. During embryonic and fetal stages of cardiac development, when the valve leaflets are still forming and the valve tissue extracellular matrix (ECM) is developing, biomechanical forces are essential to guide leaflet tissue form and composition. Biomechanical disruptions, for instance, due to abnormal blood flow conditions, can lead to malformed valve leaflets with impaired function. Yet, we do not completely understand how biomechanics guides leaflet formation.

Mechanical stress is known to actively regulate leaflet anatomy and structural composition during valvulogenesis [9,10,11]. Moreover, valve function is governed by a host of geometric parameters as well as leaflet material properties [12]. However, studies addressing structure-function relationships during heart development are scarce. Previous developmental studies have focused on the biomechanics of cardiac cushions, which are the precursors of valve leaflets, or the transition from cushions to leaflets [13,14,15]. The mechanics of the fetal aortic valve, when leaflet ECM is still forming in preparation for birth, remain largely unexplored.

Among animal models of cardiac development, the chick embryo provides an advantageous experimental model for valve formation research due to its external development, allowing direct access for advanced imaging [10,16,17]. In the chick embryo, hemodynamic and cardiac function parameters can be measured non-invasively using echocardiography, providing quantitative in vivo data essential for computational modeling [11,18,19]. Moreover, histology or micro-computed tomography (CT) imaging can provide information on leaflet anatomy. Among anatomical characteristics, the leaflet midline curve or “belly curve” was found to be a critical determinant of valve biomechanics [20,21]. Previous studies of the adult valve anatomy have shown that variations in the belly curve alter stress distributions, with changes in curvature leading to redistribution of von Mises, tensile, and compressive stress across the leaflet [21,22]. Moreover, anatomical leaflet modifications directly influence hemodynamic performance by affecting geometric orifice area (GOA), coaptation dynamics, and transvalvular pressure gradients [20,23]. Curvature-induced alterations in leaflet motion modify jet characteristics and wall shear stress patterns, stimulating mechanobiological pathways that link anatomy to cellular responses and tissue remodeling [24,25]. However, the role of leaflet anatomy during cardiac development, when embryonic valve layers are still developing, and cardiac hemodynamics is distinct from adult blood flow, is not understood.

To investigate intricate structural-functional relationships in valve leaflets, finite element analysis (FEA) has been extensively employed to model adult and prosthetic valves. FEA has been used to quantify aortic valve leaflet deformation, estimate leaflet stresses, and guide prosthesis and repair design under physiological and pathological loading conditions [26,27]. Studies include pure biomechanical (structural) FEA with prescribed pressure loads to determine transient structural response and design-level stress estimates [28,29] as well as fluid–structure interaction (FSI) [30] models that account for the dynamic interaction of the valve leaflets with blood flow. While several studies have addressed the biomechanics of adult valve leaflets, developing valve leaflets have not been modeled before.

Here, we analyzed the biomechanics of chick embryo developing aortic valves using FEA methodologies typically employed for biomechanical analysis of adult native and prosthetic valves [31,32,33,34]. For our studies, we chose embryos at the Hamburger–Hamilton [35] stage 40 (HH40—about 14 days of incubation out of 21 days to hatching), since at this stage the heart is fully formed and aortic valves are developing their ECM structure and maturing. HH40 corresponds to the third trimester of fetal development in humans. Using a parametric model of the HH40 chick embryo aortic valves, similar to parametric models used for human valve leaflets, we investigated the influence of leaflet stiffness and the belly curve on leaflet deformation and stress distribution during systole, when the valve leaflets open to allow blood to flow through the aorta. Our modeling framework enabled detailed analysis of displacement patterns, effective stress distribution, and kinematic behavior, while also providing new insights into how anatomical and material variations shape the mechanical environment of developing embryonic valves. The primary objective of this study is to determine trends in biomechanical stress and deformation modes of developing aortic valve leaflets, and to understand how leaflet biomechanics is affected by tissue material properties and anatomy.

## 2. Materials and Methods

### 2.1. Doppler Ultrasound Measurement of Aortic Flow in Chick Embryo

**Figure 1:**
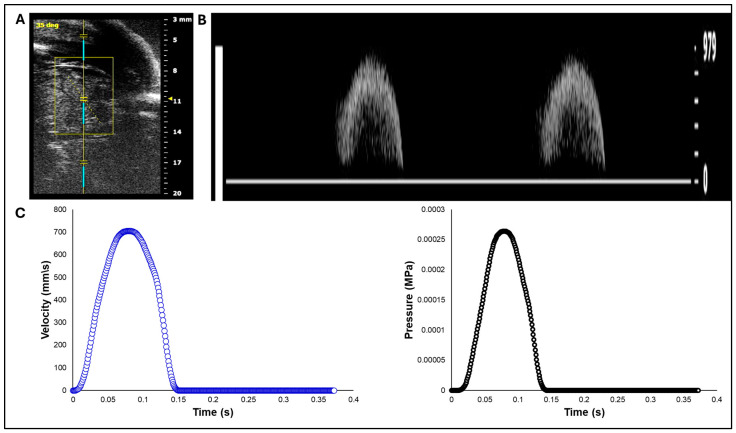
Doppler ultrasound-based acquisition and processing of aortic velocity over the cardiac cycle from HH40 (Day 14) chick embryo. (**A**) Imaging setup and location of the Doppler sample volume (gate) positioned distal to the aortic valve, with an insonation angle of about 35 degrees. (**B**) Pulsed-Wave Doppler velocity waveform. (**C**) Aortic blood flow velocity from a single cardiac cycle extracted from the Doppler waveform (left) and its corresponding pressure–time profile obtained using the simplified Bernoulli equation (right), used as a load boundary condition in FEA.

White leghorn chicken eggs were incubated for about 14 days to the Hamburger–Hamilton [35] avian developmental stage 40 (HH40). Chick embryos were imaged in ovo by creating a small window in the eggshell to expose the chorioallantoic membrane and underlying cardiovascular structures. In vivo imaging was performed with high-resolution ultrasound using the Vevo 2100 system (FUJIFILM VisualSonics Inc., Toronto, ON, Canada) equipped with an MS550S-0018 transducer (center frequency: 40 MHz, axial resolution: ~40 µm; FUJIFILM VisualSonics Inc., Toronto, ON, Canada). Long-axis B-mode images of the left ventricle and ascending aorta were acquired to guide Doppler measurement. Pulsed-Wave (PW) Doppler mode was used to assess blood flow velocity, with insonation angle maintained below 60° to minimize angular error in velocity estimation (Figure 1A). Recorded velocity–time waveforms (Figure 1B) were converted to pressure-time profiles using Bernoulli’s equation to apply as a load boundary condition in FEA (Figure 1C). The derived pressure-time profile represents an approximate systolic load specific to the HH40 embryonic circulation, which operates at significantly lower pressures than the adult human cardiovascular system. Although the simplified Bernoulli equation neglects viscous losses and flow acceleration, it is frequently used for estimating transvalvular pressure gradients [36].

### 2.2. Cryostat Sectioning

A heart, carefully dissected from an HH40 chick embryo, was arrested in the diastolic phase by immersion in cold phosphate-buffered saline (PBS) containing 10% potassium chloride (KCl). Subsequently, the heart was carefully positioned and embedded in optimal cutting temperature (OCT) compound, and flash-frozen on crushed dry ice.

After equilibrating to −20 °C within a cryostat (LEICA CM1860, Leica Biosystems, Nussloch, Germany), the tissue block was serially sectioned at a thickness of 8–10 µm. The sections were mounted on positively charged slides (Superfrost Plus™, Epredia, Portsmouth, NH, USA). For morphological visualization, the sections were subjected to a standard Hematoxylin and Eosin (H&E) staining protocol.

**Figure 2:**
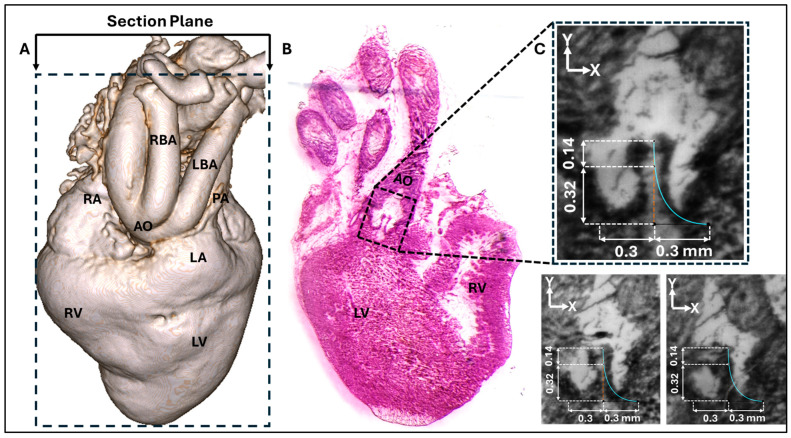
Images of the chick embryo heart at HH40. (**A**) A three-dimensional (3D) micro-CT image of the HH40 heart showing the approximate cryostat section plane location and anatomical landmarks, including the right ventricle (RV), left ventricle (LV), left atrium (LA), aorta (AO), right atrium (RA), right brachiocephalic artery (RBA), and left brachiocephalic artery (LBA). (**B**) A representative cryostat section image. (**C**) Three registered cryostat sections, each showing the aortic valve leaflet profile. The belly-curve profile was extracted from each of the three images and subsequently averaged. The resulting mean profile is displayed as a blue line overlaid on the three sections for comparison.

Stained sections were imaged using a light microscope (LEICA M205 A, Leica Microsystems, Wetzlar, Germany) equipped with a digital camera (sCMOS pco.edge 5.5; PCO AG, Kelheim, Germany). The resulting digital images were used to analyze the aortic valve leaflets for modeling (Figure 2).

### 2.3. Aortic Valve Modeling

**Figure 3:**
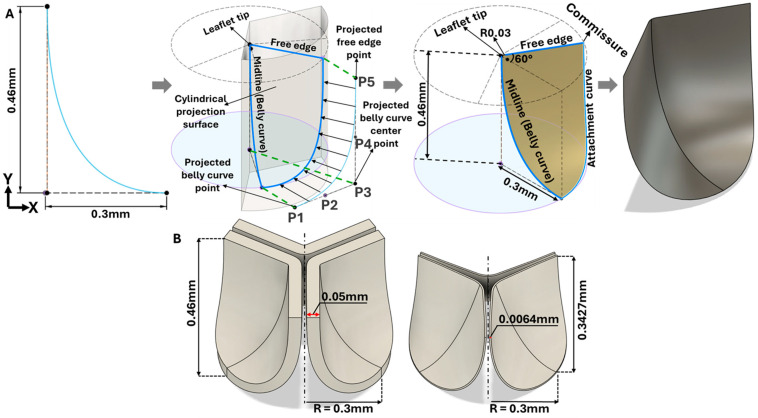
Geometric construction of the HH40 aortic valve parametric model. (**A**) 3D modeling steps based on the belly curve (middle leaflet curve) extracted from cryostat images. By construction, the belly curve divides each leaflet into two identical halves but also defines a region of elevated stress, as the curvature along this line becomes much larger than in the rest of the leaflet. (**B**) Comparative visualization of chick embryo (left) and human (right) aortic valves scaled to the same annulus radius (0.3 mm).

Three representative consecutive cryostat sections that clearly show the HH40 aortic valve leaflets (Figure 2B,C) were selected for measurement and parametric valve design. The images were registered and calibrated using ImageJ (version 1.54p; National Institutes of Health, Bethesda, MD, USA) software. Subsequently, the calibrated images were imported into Autodesk Fusion (version 2.0.21508; Autodesk Inc., San Francisco, CA, USA) and positioned on the x–y plane. The images were aligned so that the annulus plane and the centerline were coincident. The leaflet curve profiles on each image were drawn using a Bezier curve based on three reference points (upper, middle, and outer points, see Figure 2C and Figure 3A) that were localized (and aligned) on the image x–y plane. A representative curve was then obtained by averaging each point group (upper, middle, and outer) and generating an average leaflet midline (belly curve) profile (displayed in Figure 2C for each image). Based on the location of the average points and imaging data, the valve height and annulus radius were 0.46 mm and 0.3 mm, respectively (see Figure 2C). To avoid geometric inconsistencies (overlapping) during reconstruction of the valve’s three leaflets, the axis of the belly curve was shifted by 0.005 mm in the x-direction, generating a small gap (0.01 mm) between the leaflets in the reconstructed geometry (Figure 3A). Thus, the three points of the Bézier curve defining the belly curve were as follows: upper (0.005 mm, 0.46 mm); middle (0.005 mm, 0 mm); and outer (0.3 mm, 0 mm). The leaflet free edge was created by rotating the x–y plane containing the belly curve 60 degrees counterclockwise, at the intersection with a perpendicular plane at y = 0.46 mm (see Figure 3A). Finally, the belly curve and free edge were connected by an attachment curve, generated by projecting a five-point Bézier curve onto a cylindrical surface created by extruding the annulus circle along the y-axis (Figure 3A). The five points x, y, and z coordinates in mm were as follows: (P1: 0.473, 0.10, 0.00); (P2: 0.398, 0.23, 0.00); (P3: 0.324, 0.358, −0.001); (P4: 0.324, 0.359, 0.203); (P5: 0.323, 0.36, 0.46). Through this construction, the geometric contours of half of the aortic valve were completed. A surface was generated over the resulting sketches using the surface patch method. This half-valve leaflet was then mirrored through the x–y plane (the plane of the belly curve) and tripled around the central axis using a circular pattern to form the complete aortic valve (consisting of three identical leaflet surfaces). Finally, the generated surfaces were converted into solid geometry using the average valve thickness (0.05 mm) measured from cryostat images.

### 2.4. Middle Curve Shift-Based Parametric Modeling of Aortic Valve Leaflets

**Figure 4:**
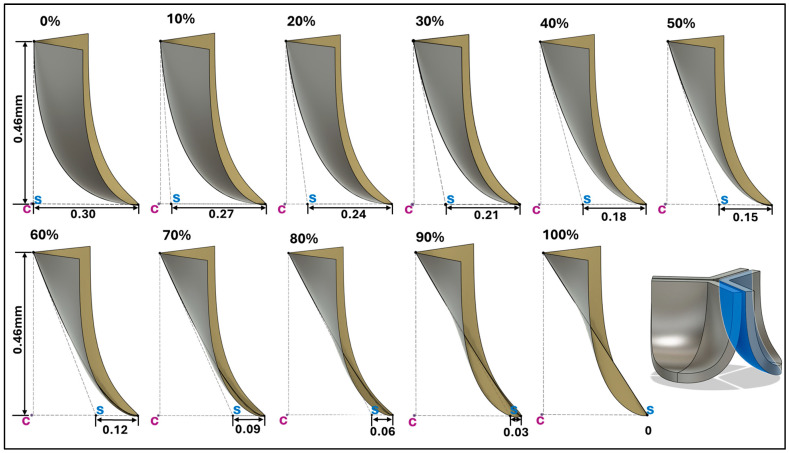
Generation of distinct aortic valve leaflet anatomies. The parametric leaflet geometries were altered by systematically displacing the middle point (“C”, purple) of the three-point Bézier curve representing the belly curve (“S”, blue) toward the maximum annulus radius. The displacement was performed in 10% increments from 0% to 100%. A total of 11 leaflet surface geometries were generated: six (0–50%) in the top row and five (60–100%) in the bottom row. At the end of the second row, a 3D model of the aortic valve is also shown, with the highlighted blue surface corresponding to the leaflet region depicted in all 11 geometries.

To determine how valve anatomy affects leaflet biomechanics, we generated 11 distinct leaflet profiles by parametrically changing the belly line curve. To this end, we shifted the middle point of the Bézier curve representing the midline curve in increments of 0.03mm in the belly-curve plane x-direction (see Figure 4), corresponding to 10% increments. This resulted in a transition from the native (measured) curved to a nearly linear belly-curve profile. All other parameters used in the construction of the leaflet anatomy were kept the same. Note that with increasing middle point shift, marked changes in leaflet curvature are accompanied by an increase in the leaflet attachment angle to the aortic wall (from 0 to about 57 degrees), which affects stress concentrations along the belly curve.

### 2.5. Finite Element Analysis Setup

**Figure 5:**
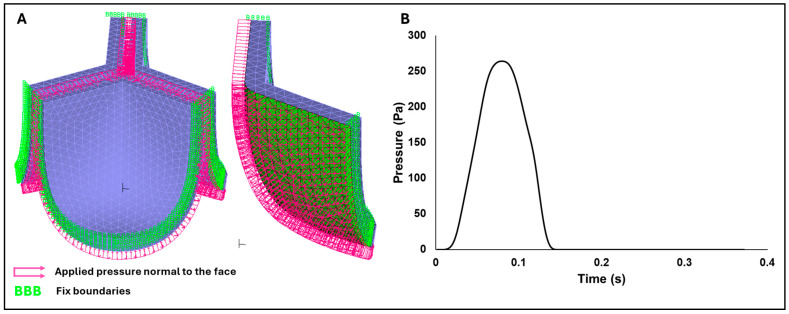
FEA boundary conditions. (**A**) Visualization of the applied boundary conditions on the aortic valve model, including frictionless face-to-face contact (green inner surfaces) and fixed side constraints (also in green). (**B**) Transient pressure–time profile applied normal to the inner (ventricularis) valve surfaces and used as the loading condition in the finite element simulations.

Dynamic implicit finite element simulations were performed in ADINA software (version 24.0; Bentley Systems, Inc., Exton, PA, USA), incorporating large-displacement and large-strain formulations to accurately capture the nonlinear structural response of the valve leaflets. Frictionless face-to-face contact was defined on the inner (ventricularis) leaflet surfaces, while side boundaries were fixed (to the outside cylindrical shape). Moreover, a transient pressure profile was applied normal to the leaflet ventricularis surface (pink arrows in Figure 5A with the time-dependent pressure profile in Figure 5B). The simulations were performed using a maximum time step of 0.001 s and the ADINA automatic time stepping (ATS) method, which adaptively reduces the time step size to facilitate convergence when a solution fails to converge. Finite element analysis (FEA) was initially performed to determine leaflet mechanical properties, and then for the eleven valve geometries with belly-curve shifts (0–100%; Figure 4). Each simulation was run for 0.155 s, corresponding to the effective duration of the HH40 chick embryo cardiac systolic phase. Therefore, simulations included the opening of the valve followed by partial closure (to the initial configuration). Due to the absence of retrograde pressure data during the late systolic and early diastolic phases, full valvular coaptation and the associated contact mechanics were not simulated.

### 2.6. Mesh Generation and Convergence

The FEA leaflet model was meshed with tetrahedral elements generated on the default (non-shifted) valve geometries using HyperMesh NVH (Student License, Version 2024; Altair Engineering Inc., Troy, MI, USA). For mesh convergence analysis, four different mesh configurations were created using the following settings: (1) 28,902 nodes and 17,990 elements with an element size of 0.03 mm and a growth ratio of 1.2; (2) 35,730 nodes and 32,578 elements with an element size of 0.025 mm and a growth ratio of 1.2; (3) 74,304 nodes and 49,071 elements with an element size of 0.021 mm and a growth ratio of 1.2; and (4) 99,858 nodes and 66,545 elements with an element size of 0.019 mm and a growth ratio of 1.2. To ensure numerical stability and mesh quality across all geometries, strict quality criteria were enforced: the tetrahedral collapse value was maintained above 0.3, while element aspect ratio (<5) and skewness (<0.85) were monitored during simulations.

Mesh convergence was tested twice. First, a preliminary mesh convergence analysis was performed using human aortic valve material properties from the literature (see Table 1, Test 0). Using the mesh derived from this initial convergence analysis, a material sensitivity analysis was performed with distinct material parameters (see Section 2.7) to determine suitable material parameters for the chick aortic leaflets. Once material parameters were selected from the sensitivity analysis, a second mesh convergence analysis was performed. The resulting mesh and material properties were used in FEA to simulate the biomechanical effects of changing the belly curve of the chick aortic valve leaflets through a progressive shift in the parametric model. To determine mesh convergence, maximum displacement values at the valve leaflet tip were used. This metric was selected because the leaflet tip, which exhibits the largest deformations, is also the most sensitive to biomechanical load and the accuracy of the solution.

### 2.7. Material Sensitivity Analysis

The material properties of the HH40 chick embryo aortic valve have not been defined in the literature. In the absence of direct experimental data, we adopted an Inverse Finite Element Analysis (iFEA) approach. Following methodologies described by Aggarwal and Sacks, 2016 [37], we inferred material properties from the functional requirement of maximizing GOA during systole without prolapsing (tip leaflet displacement < aortic annular radius), assuming native tissues are optimized for flow efficiency. Hence, we adopted a multi-stage methodology to estimate the material properties of valve leaflets. While adult valve tissue typically exhibits anisotropy due to collagen fiber alignment, the microstructural orientation in HH40 embryonic valves is not yet fully characterized, and likely not fully developed. However, recent computational studies have demonstrated that isotropic Ogden models can successfully capture global valve kinematics and hemodynamics with high accuracy compared to anisotropic models, while capturing strain-stiffening behavior [38,39]. Therefore, the isotropic Ogden hyperelastic model was selected as a robust phenomenological approximation, consistent with standard biomechanical modeling practices for soft tissues where specific fiber data is unavailable [40]. Material model parameters were then refined through a material sensitivity analysis aimed at maximizing GOA, which was used as a proxy criterion for determining chick embryo aortic valve material properties.

The Ogden strain-energy function, W, has the following form:

(1) $$W(\lambda_{1},\lambda_{2},\lambda_{3})=\sum_{p=1}^{N}\frac{{µ}_{p}}{\alpha_{p}}\left({\lambda_{1}}^{\alpha_{p}}+{\lambda_{2}}^{\alpha_{p}}+{\lambda_{3}}^{\alpha_{p}}\;-3\right)$$

where $\lambda_{i}$ are the principal components of the stretch tensor, and *N*, ${µ}_{p}$ and $\alpha_{p}$ are material constants. For small deformations, the shear modulus $G_{0}$ can be approximated as follows [40]:

(2) $$G_{0}\approx \frac{1}{2}\sum_{p=1}^{N}{µ}_{p}\alpha_{p}$$

Following previous works [41], we used N = 1. For human leaflet tissues, with μ_1_ = 577.6 Pa, and α_1_ = 26.26, rendering $G_{0}=7584$ Pa. For the chicken embryo, we found material parameters for a much earlier developmental stage (HH12, Day 2), when the valve leaflets are not yet formed [42]. However, the study characterized Ogden material properties for the myocardium and cardiac jelly (the latter giving rise to valve leaflets later during development), with initial shear modulus (G_0_) of about 80 Pa and 27 Pa, respectively. The HH40 aortic valve, the focus of our study, is expected to be significantly stiffer than this early-stage cardiac tissue [42]. The parameters selected for Test 10 (μ_1_ = 105 Pa, α_1_ = 10), the lower bound for leaflet material properties in our sensitivity analysis, yield G_0_ ≈ 525 Pa. This value was chosen as a conservative lower bound for the HH40 aortic valve, representing a stiffness about 20 times greater than the cardiac jelly at HH12 [42] to reflect the expected developmental stiffening. The other values (Tests 1–9) were linearly interpolated between the upper bound from Test 0 (based on human aortic valve data [41]) and the lower bound from Test 10 (Table 1).

**Table 1:** Ogden hyperelastic material parameter sets were used in sensitivity tests, since HH40 (Day 14) chick embryo aortic valve properties were not available. Test 0 corresponds to human adult leaflet properties [41], and test 8 (bold) was chosen here for the HH40 aortic valve leaflets (see Section 3.1).

TEST	*μ*_1_ (Pa)	*α*_1_	$G_{0}$ (Pa)
0	577.60	26.260	7583.89
1	530.34	24.634	6532.20
2	483.08	23.008	5557.35
3	435.82	21.382	4659.35
4	388.56	19.756	3838.20
5	341.30	18.130	3093.88
6	294.04	16.504	2426.42
7	246.78	14.878	1835.80
**8**	**199.52**	**13.252**	**1322.02**
9	152.26	11.626	885.09
10	105	10	525.00

### 2.8. Frequency Analysis and Damping Coefficient Calculation

When analyzing the dynamic behavior of structural systems, undamped vibrations near resonance modes can result in unrealistic solutions. Typically, vibrations are physically dampened (at least to some extent) by natural energy dissipation mechanisms. To suppress non-physical vibrations, frequency analysis is often performed to identify the natural frequencies of the system and determine appropriate Rayleigh damping coefficients [43]. For a given vibration mode, the modal damping parameter ($\xi_{i}$) can be expressed in terms of the Rayleigh damping coefficients α and β as follows:

(3) $$\xi_{i}=\frac{\alpha }{{2\omega }_{i}}+\frac{\beta \omega_{i}}{2}\;\;\;\;\;$$

where $\omega_{i}$ is the natural angular frequency of mode *i* in rad/s, α represents the mass-proportional damping coefficient, which predominantly damps lower modes, whereas β is the stiffness-proportional damping coefficient, which primarily targets higher modes. Unless high-frequency damping is specifically needed, it is generally recommended to set β = 0 or assign a very small value, especially if explicit analysis is performed (as β can severely reduce the critical time step for time stability) [44]. In this project, we performed frequency analysis of the valve leaflet geometry and used the lowest natural frequency to determine the Rayleigh damping coefficient (α) based on different damping levels. Given distinct α values, we then tested the oscillation of the valve leaflet under the pressure load (Figure 5B) and selected the minimum damping required to avoid unwanted leaflet oscillations.

## 3. Results

### 3.1. Mesh Convergence and Material Sensitivity

**Figure 6:**
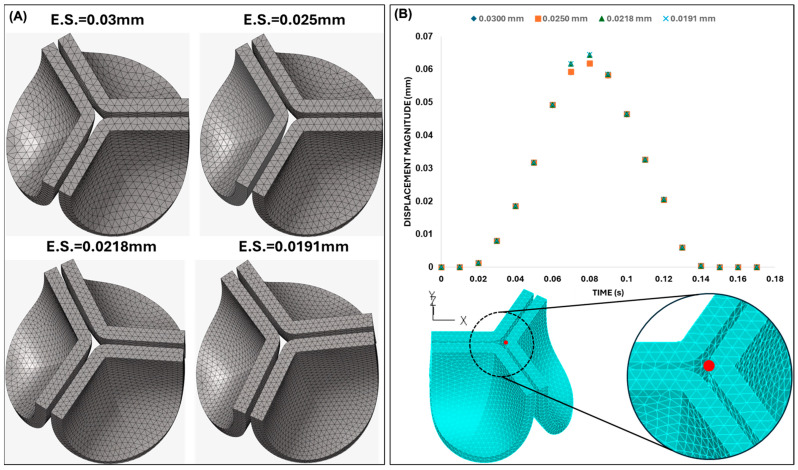
Initial mesh convergence analysis. (**A**) Tetrahedral mesh configurations generated on the default (non-shifted) valve geometry with four different element sizes (ES): 0.030 mm, 0.025 mm, 0.0218 mm, and 0.0191 mm. (**B**) Time-dependent displacement magnitude at the tip of the valve leaflet computed during initial mesh convergence analysis. The measurement point (leaflet tip) is indicated in red on the aortic valve mesh shown below the plot.

An initial mesh convergence analysis was performed using the material properties corresponding to the adult human valves. Four mesh sizes were evaluated by plotting the displacement of the leaflet tip over time (see Figure 6) when the aortic pressure load was applied. We selected the mesh with an element size of 0.025 mm, as the difference in the tip displacement with respect to the finer mesh (with element size 0.0191) was within 4%.

**Figure 7:**
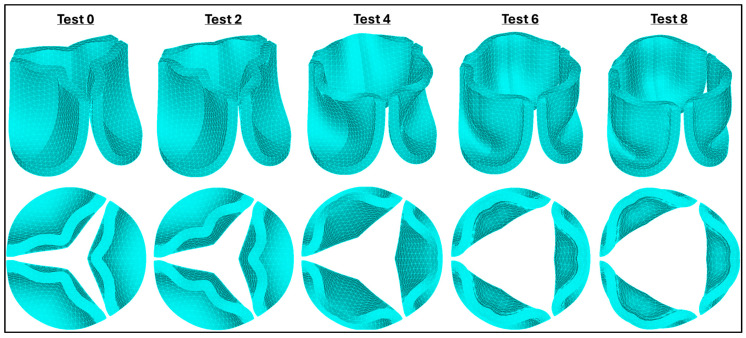
Valve leaflet configurations at peak blood pressure (t = 0.08 s). Columns indicate progressively softer (less stiff) material properties, starting from adult human leaflet properties (from [41]). (**Top**): perspective views of the aortic valve. (**Bottom**): top view of valve leaflets for selected material cases tested during sensitivity analysis. The comparison illustrates the effect of varying material properties on valve opening and leaflet displacement.

Using the selected mesh (size 0.025 mm), we conducted a leaflet material sensitivity analysis. To this end, we applied the aortic pressure load previously estimated from aortic velocity measurements (Figure 5B). We then examined the valve configuration at 0.08 s, which corresponds to the time point of peak aortic pressure in the HH40 chick embryo, as progressively softer leaflet material properties were simulated (see Figure 7). As the parameters in the Ogden strain-energy function progressively decrease, valve leaflet stiffness decreases, and the valves open more, until the leaflets become fully open. The optimal material model selected (μ_1_ = 199.52 Pa, α_1_ = 13.252) was based on two criteria: (1) achieving the largest effective GOA, and (2) ensuring that the maximum leaflet tip displacement remained within the annular diameter of the valve to avoid leaflet prolapse.

A second mesh convergence analysis was then performed before proceeding with further analysis. The same four mesh configurations employed previously (Figure 6A) were used in this new convergence analysis, and we required a difference of <1% in tip displacement (at peak pressure, t = 0.08 s) from progressively finer meshes as a criterion for convergence. Based on our results, the mesh with an element size of 0.025 mm (with 0.06% difference with respect to the displacement of the 0.0191 mm element size mesh) was again selected as an optimal mesh for our studies.

**Figure 8:**
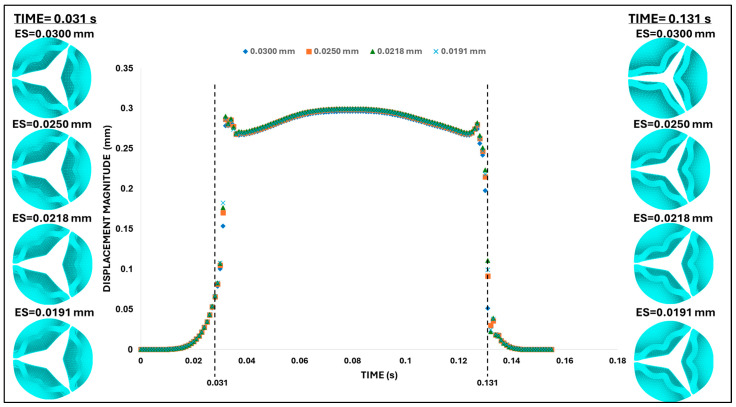
Aortic valve configuration and leaflet tip displacement during the second (final) mesh convergence analysis. Top-view comparisons of aortic valve deformation patterns for different element sizes (ES = 0.030 mm, 0.025 mm, 0.0218 mm, and 0.0191 mm) during the transition phases. Left column: valve leaflet deformation during the opening phase (t = 0.031 s). Right column: corresponding deformation during the closing phase (t = 0.131 s). Center: time-dependent leaflet tip displacement magnitude for all element sizes used to evaluate convergence behavior. Mesh refinement beyond 0.025 mm produced negligible overall changes in displacement/configuration over the cardiac cycle and was used in further analyses.

At peak pressure, the displacement of the leaflet tips was almost insensitive to the mesh element size, easily satisfying our mesh convergence criteria. During the rapid opening and closing phases, tip displacements were more sensitive to the element size. To examine this behavior, two characteristic time points were selected: (i) the valve opening phase (t = 0.031 s) and (ii) the valve closing phase (t = 0.131 s). Both times correspond to the middle of the opening and closing transition periods (see Figure 8). These are highly dynamic instants within the cardiac cycle, for which precise leaflet motion is difficult to characterize. While differences in leaflet configuration and tip displacement were negligible during opening (t = 0.031 s), they were evident during closing (t = 0.131 s) but quickly stabilized to similar values upon further closing (t > 0.031 s). Comparing average errors over the systolic phase (with respect to the finer mesh) demonstrated that the mesh with element size 0.025 mm (with 0.7% average error) provides a balance between computational efficiency and physical accuracy.

### 3.2. Damping Coefficient Sensitivity

The fast valve opening and closing generate oscillations in the leaflet motion (see Figure 8). To damp these oscillations, mass-proportional damping coefficients (α, see Equation (3)) were calculated based on the first-mode angular frequency of the aortic valve, $\omega_{1}$ = 4779.47 rad/s, so that the modal damping parameter, ξ, ranged from 0.001 to 0.2 (the latest corresponding to 20% damping, see Table 2). These α values were then used to conduct a damping sensitivity analysis, employing the same element size and material properties determined in the mesh and material convergence analyses.

**Figure 9:**
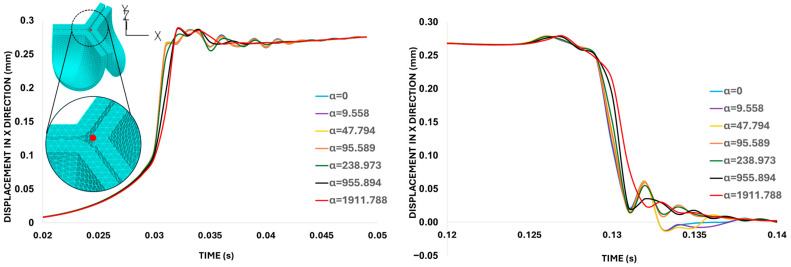
Damping sensitivity analysis. Displacements in the X-direction (on the belly-curve plane) quantified for the leaflet tip (red-marked point on the aortic valve leaflet) during valve opening and closing, simulated with different mass-proportional damping values (α). The left graph shows the valve-opening response (between t = 0.02 s and 0.05 s), while the right graph focuses on the valve-closing interval (between t = 0.12 s and 0.14 s). Both graphs indicate that the system is effectively damped when α = 1911.7880 s^−1^ (highlighted in red), corresponding to 20% critical damping.

The leaflet tip point displacement results in Figure 9 indicate that the mass-proportional damping value of α = 1911.79 s^−1^ (corresponding to 20% critical damping ratio) successfully damped excessive valve motion. Therefore, this specific α value was selected for the subsequent analysis of the aortic valve.

### 3.3. Valve Leaflet Middle Curve Shift

The belly curve is a critical anatomical valve feature that controls valve-opening kinematics, GOA, and coaptation potential. To address the biomechanical question of how leaflet curvature dictates the trade-off between tissue loading and valve-opening efficiency, we performed FEA for the eleven valve geometries with varying belly curve shifts (0–100%). To this end, we utilized the leaflet mesh and material properties determined before (element size: 0.025mm; Ogden parameters: µ1 = 199.520 and α1 = 13.252; and Rayleigh damping coefficient: α = 1911.79) and applied the time-dependent systolic blood pressure profile determined previously (Figure 5B). Simulated valve displacement and effective stress contour plots, as well as the valve configuration during opening (ascending transition) and at peak pressure, are shown in Table 3. Examination of the leaflet configuration during opening and peak pressure reveals that the tip displaces faster than the valve base. Interestingly, this behavior could be due to the pressure boundary conditions applied simultaneously to the entire valve ventricularis surface. Not surprisingly, since displacements are higher at the leaflet tip (top of the middle leaflet curve), effective stresses are also higher in this region. At 0% shift, broader regions of the leaflets exhibit high displacement; however, this distribution gradually decreases up to the 20% shift and nearly disappears at 30%. Beyond 30%, the previously wide high-displacement regions become localized near the leaflet tip, and, between 70% and 100% shift, they are confined to smaller areas of the leaflet tips. Thus, the increasing linear geometry of the belly curve also restricts leaflet motion.

As expected, effective stress plots show that regions of high displacement are often accompanied by elevated effective stress concentrations (≥0.015 MPa, see Table 3), particularly in the upper belly curve region. Moreover, stress plots show a progressive redistribution and mitigation of stress with increasing belly-curve shifts. Concurrently, as the shift increases from 0% to 100%, the regions of maximum effective stress (≥0.015 MPa) become progressively localized at the commissure edge (the joining edge between two valve leaflets).

**Figure 10:**
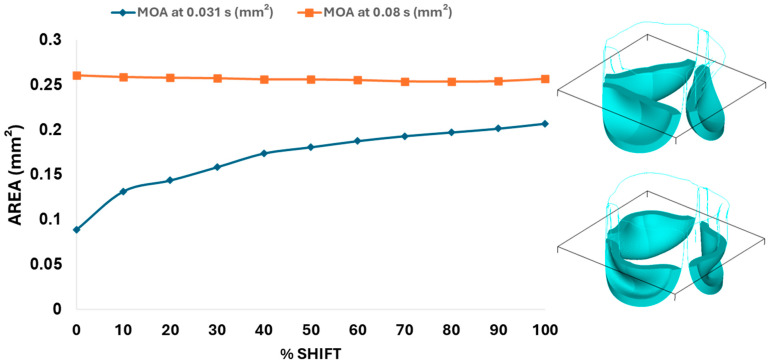
Middle orifice area (MOA) variation over time for different belly-curve-shifted valve geometries. The blue curve represents MOA measured at the ascending transition phase (t = 0.031 s), and the orange curve represents MOA at the peak pressure (t = 0.08 s). The right panel shows the plane used for MOA calculation, with the upper image depicting the valve cross-section at t = 0.031 s and the lower image showing the cross-section at t = 0.08 s.

Next, we determined the valve middle orifice area (MOA), on a plane orthogonal to the valves and located halfway between the base and the tip of the valve leaflet (z = 0.23 mm, see Figure 3A and Figure 10), both during valve opening (t = 0.031 s) and peak pressure (t = 0.08 s). During valve opening, the MOA increases with belly-curve shift. In contrast, the MOA at peak pressure exhibits a slight but consistent decrease with increasing belly-curve shift (Figure 10).

**Figure 11:**
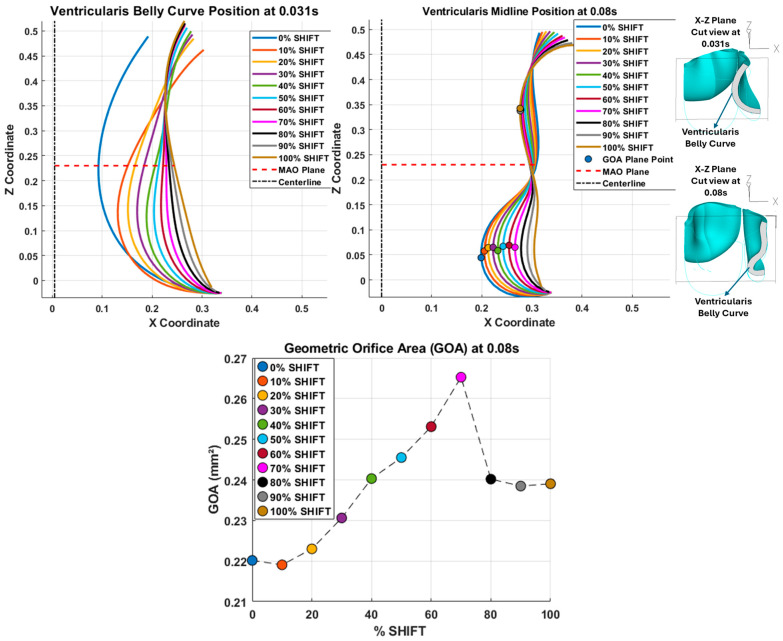
Ventricularis belly-curve profiles of the aortic valve leaflet and resulting geometrical changes. **Top-left** panel: midline leaflet deformation at t = 0.031 s (valve opening). **Top-right** panel: superimposed belly-curve profiles at t = 0.08 s during peak pressure. On each midline profile, a color-matched circular marker indicates the point closest to the centerline. **Bottom** panel: geometric orifice area (GOA) as a function of the belly-curve shift percentage. In the upper panels, the red dashed lines indicate the location of the mid-orifice area (MAO) plane (see Figure 10), and circular markers indicate the variable GOA planes.

Examining the ventricularis belly curve profiles of the aortic valve leaflet (Figure 11) reveals how the shift alters the leaflet motion. As the shift increases, the total length of the belly curve decreases, and the attachment angle to the aortic wall increases, also affecting leaflet motion and stresses. Analysis of the GOA (see Figure 11, bottom panel) reveals a nonlinear trend. Unlike MOA at t = 0.08 s (Figure 10), which decreases slightly, the GOA at t = 0.08 s (Figure 11), after slightly decreasing from 0 to 10% shift, progressively increases and reaches a maximum at 70% shift, but then reduces as the shift further increases. This trend is directly linked to leaflet deformation shape at peak pressure load (see Figure 11). From 0 to 70% shift, the GOA plane remains stable near z = 0.05 mm. However, from 80% to 100% shift, the GOA plane switches positions to z = 0.35 mm. This switch in the GOA plane, due to the leaflet deformation profile, results in a sudden drop in GOA at 80% to a value nearly identical to that of the 40% shift, which then holds steady through the 100% shift.

**Figure 12:**
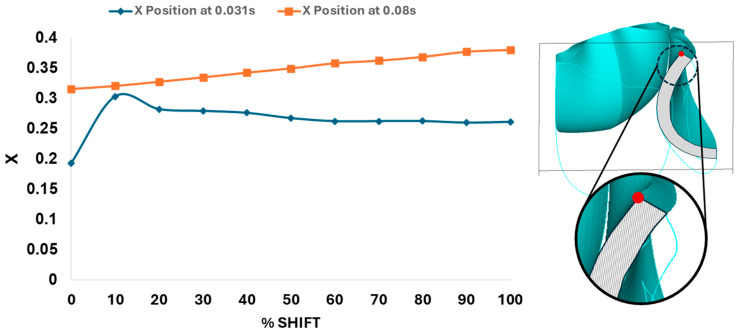
Leaflet tip displacement from the valve centerline axis (x-direction in the x–y midline curve plane) versus belly-curve shift. The leaflet tip is indicated by the red dot on the rightmost panel. The graph shows the tip position at t = 0.031 s and t = 0.08 s, corresponding to the ascending transition and peak pressure phases of valve opening, respectively.

Dynamically, at 0% shift, the valve leaflet is the slowest to displace during valve opening, and its base restricts the effective orifice area. In contrast, during aortic valve opening, the shorter length of the belly curve at 100% shift and its increased attachment angle allow it to displace faster but at the expense of increased tip bending during peak pressure (see Figure 11). During the valve-opening phase (t = 0.031 s), the valve tip closely followed the curvature of the belly curve without noticeable bending. However, at the peak pressure phase (t = 0.08 s), the leaflet tip exhibited progressive linear bending as the belly-curve shift increased from 0% to 100%, reaching nearly 90° at 100% shift. Moreover, the position of the tip changes substantially with the belly-curve shift (Figure 12).

**Figure 13:**
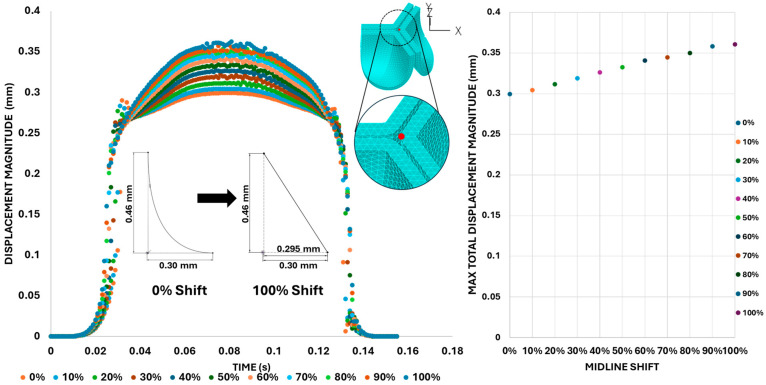
Summary displacement analysis results across different visualization levels. (**Left**): displacement magnitude–time curve color-coded by percentage belly-curve shift. The plot also shows the belly-curve shift profile and location of the tip point (red dot on upper plot). (**Right**): maximum tip displacement values as the belly-curve shifts from 0% to 100%.

Figure 13 presents the time-dependent displacement magnitude at the uppermost point (tip) of the leaflet belly curve. Note that oscillations develop after the rapid opening phase and then in the region of maximum pressure as the shift increases. At t = 0.08 s, when the maximum pressure acts on the leaflet, the tip displacement profile exhibits a nearly linear increase from 0% to 100% shift (R^2^ = 0.9953, Figure 12). At t = 0.031 s, when the valve is opening, there is a significant variation in the tip position (see also Figure 11 and Figure 12).

**Figure 14:**
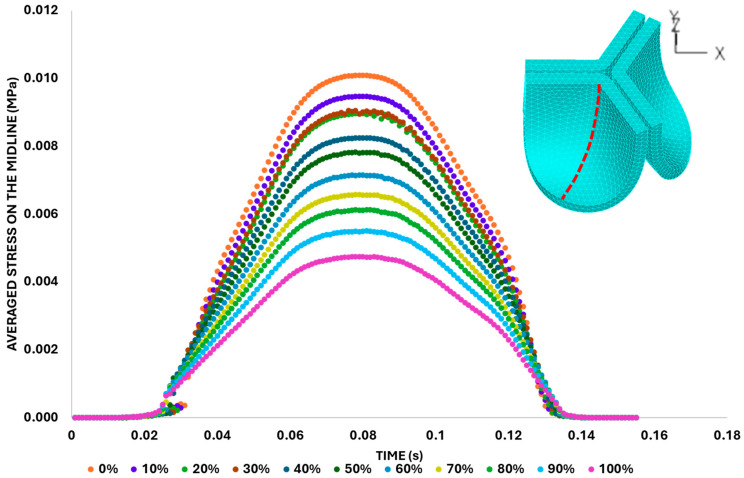
Average effective stress along the fibrosa surface of the aortic valve for eleven belly-curve-shifted geometries (0–100%). The measurement line is indicated by red dashed lines, and the corresponding average stress values at peak pressure (t = 0.08 s) are shown on the right-side graph.

We then quantified the average effective stress along the belly curve on the fibrosa surface (highlighted by the red dashed lines in Figure 14). At the peak pressure (t = 0.08 s), the 0% shift configuration exhibits the highest average effective stress while the 100% shift shows the lowest (Figure 14). Overall, we found a linear decrease in the belly curve average stress as the belly curve shift increases from 0% to 100% (R^2^ = 0.9875).

## 4. Discussion

This study investigated the biomechanics of developing aortic valve leaflets. We extracted geometrical data from HH40 chicken embryo developing valves from imaged frozen sections of the heart and used parametric analysis to reconstruct the 3D leaflet anatomy. We then tested different leaflet material properties in silico, starting with measured human adult leaflet nonlinear properties and progressively reducing the stiffness until the leaflets, subjected to pre-determined pressure load (approximated from aortic flow measurements in the embryo), completely open under the applied pressure profile. Finally, we investigated the effect of valve leaflet anatomy by progressively changing the leaflet middle curve (belly curve) and quantifying systolic biomechanical changes (displacement, stress) while keeping the leaflet properties constant. To our knowledge, this is the first study that investigates the biomechanics of HH40 chick embryo valve leaflets, including characteristic deformation modes. While we found that leaflet biomechanics is sensitive to anatomy and material properties, which need to be carefully determined, the trends obtained here are useful to better understand valve biomechanics and their dynamic behavior under blood flow.

### 4.1. Limitations

The strength of our study is the methodological approach, which was designed to estimate leaflet material properties and investigate deformation modes of developmental valve leaflets while assessing how these modes are affected by valve anatomy, with a focus on the role of the belly curve. Limitations of this study are mainly associated with the lack of data on the HH40 valve function and tissue properties. This lack of data is partly due to the tiny dimensions of the inner aortic root (<1 mm), leaflet thickness (<0.1 mm), and fast heartbeat (160–200 bpm). It is extremely difficult to image the dynamic motion of leaflets, resolve their native anatomy, and test their material properties. To overcome this lack of data, we have resorted to various assumptions as well as sensitivity analyses. We will discuss various assumptions in our study and limitations associated with them.

Achieving a perfect cryostat section precisely aligned with the true belly curve of a single leaflet is challenging, given the small size of the HH40 embryo heart and its aorta, and the manual process of orienting the tissue within the block for sectioning. Evidence of this misalignment is presented in Figure 2B,C, where two leaflets are visible in the sectioning plane; a perfectly parallel cut through a single leaflet’s belly curve would capture only one leaflet (see Figure 3). Therefore, the baseline belly curved profile used in our model should be understood as a high-fidelity approximation derived from the best achievable sections (with n = 1), rather than an exact anatomical trace. Because of uncertainties associated with our baseline curve, we adopted a parametric modeling approach and analyzed the effect of altering the belly curve. Future work should aim to create more precise 3D models of the aortic valve using non-destructive imaging techniques, such as high-resolution micro-CT imaging, that overcome the alignment limitations of physical sectioning.

The use of the simplified Bernoulli equation to estimate applied pressure loads on valve leaflets (ventricularis surface) from available aortic velocity data neglects viscous effects, which are relevant at the embryonic scale. In fact, the flow in the HH40 chick aorta is laminar. Calculating the Reynolds (Re) number using the conservative peak-velocity method [45] with our measured peak velocity of 705 mm/s and annulus diameter of 0.6 mm yields a peak Re ≈ 300. While higher than early-stage reports, this value remains well below the turbulent threshold (Re < 2000), confirming that viscous forces dominate the behavior of blood flow near the leaflets, as seen in earlier chick development [46]. Future studies incorporating FSI models of the valve, with a laminar flow assumption (that will further mitigate errors associated with turbulence [46]), will provide a more comprehensive analysis of the hemodynamic forces involved and biomechanical interactions with valve leaflets.

Unavailability of mechanical properties for HH40 chick embryo aortic leaflets prompted us to employ a sensitivity analysis to estimate leaflet material properties. Direct use of adult human material properties (or even chicken adult properties) is inadvisable, since we expect large changes in material properties as valve leaflets mature and grow from embryonic development to adult size. This expectation is supported by experimental studies on embryonic valve mechanics [47], which demonstrated a monotonic increase in leaflet stiffness at early stages (from HH25 to HH34) due to collagen organization. For our HH40 model, a significantly later developmental stage, it is biomechanically justified to assume that material properties have stiffened beyond the softer HH34 baseline, but since their ECM is still developing, mature values have not been achieved. In the absence of validated data, we adopted the principle of maximum functional efficacy: we chose material properties that allowed full opening of the leaflets under the peak applied pressure while avoiding leaflet prolapse (see Figure 7, parameter set 8 from Table 1). These optimized material properties, based on an Ogden hyperelastic model, represent a physiologically effective outcome for the given systolic peak load and anatomy. Although a dedicated pressure-sensitivity analysis was not performed, our material sensitivity analysis functionally serves a similar purpose. In FEA, the structural deformation response is a function of the stiffness-to-load ratio; therefore, testing a wide range of material stiffness effectively maps the solution space for varying pressure loads.

It is also crucial to note that embryonic valve leaflets (HH40) are anatomically distinct from adult valves: human aortic valve leaflets scaled to an equivalent annulus diameter are nearly 7.8 times thinner than HH40 chick aortic valve leaflets (see Figure 3B). The biomechanical analysis performed in our study provides a focused understanding of how the unique embryonic valve anatomy dictates opening mechanics and stress distribution under systolic pressure.

The Rayleigh damping coefficient was determined based on a damping sensitivity analysis. As a result of this analysis (Figure 9), 20% of critical damping was selected for our studies, effectively eliminating leaflet oscillations. The primary reason behind this deliberate choice was the absence of experimental data characterizing the natural vibration profile of the HH40 embryonic aortic valve. Our objective was to achieve numerical stability rather than to replicate a speculative oscillation pattern. Accordingly, leaflet vibration was intentionally minimized in the model.

Other key (yet common) simplifications in our developmental valve leaflet model are the stress-free configuration, assumed to be the initial leaflet configuration (Figure 4); the leaflet sharp change in overall curvature across the belly curve; the lack of direct contact within leaflets (with a small gap in between to avoid overlap of leaflets); and restriction of the analysis to the opening (systolic) behavior, while ignoring coaptation mechanics. It is evident that the stress and displacement fields quantified under these assumptions differ from physiological conditions. However, assumptions made in this study allowed for a consistent analysis of how changes in material properties and leaflet anatomy influence valve leaflet biomechanics. While actual stress and displacement quantifications within chick aortic leaflets differ from those reported here, trends found and general leaflet opening/closing characteristics described provide valuable insights into the aortic leaflet dynamic behavior during embryonic development.

Despite limitations, the methodology employed in this study provides a platform for testing scenarios as well as refining models as data on chick embryonic valves becomes available, and FSI models are implemented.

### 4.2. Parametric Valve Modeling

Parametric aortic valve modeling is a common method, largely because the valve’s thin leaflets are difficult to resolve in their native morphology, even for large animal models and humans [34]. This approach defines the complex, 3D anatomy of the leaflets using a limited set of controllable parameters. Adopting this methodology in the present study, the leaflets were constructed using key anatomical curves: the free edge (top of the leaflet), the attachment line, and the belly curve. The flexibility of parametric modeling is evident in the literature, where alternative functions such as hyperbolic curves [48] and definitions based on key anatomical points [49,50] have been successfully employed. This overall strategy has been extended to create unified 3D representations of the entire valve and root [34]. In this study, we specifically utilized Bézier curves to represent the leaflet belly curve (3-point Bézier) and the attachment line (5-point Bézier). The use of Bézier curves to represent leaflet geometry is a technique consistent with other contemporary approaches [51]. We used parametric valve modeling to not only generate a representation of the embryonic valve leaflets in 3D but also to alter the valve leaflet anatomy in a relatively simple way as we performed a geometric sensitivity analysis.

### 4.3. Leaflet Material Properties

The mechanical response of the leaflet tissue was described in this study using an Ogden-type hyperelastic constitutive model [40], a nonlinear isotropic constitutive model frequently employed to describe the behavior of soft tissues. While studies on prosthetic or well-characterized leaflet tissues often favor anisotropic models such as the Fung-type [49,52,53] or Holzapfel [54,55] to account for fiber orientation, the Ogden model is suitable for biological materials for which detailed microstructural data are unavailable. However, it is important to note that while the isotropic Ogden model accurately predicts leaflet kinematics and GOA, it may simplify the stress tensor compared to fiber-reinforced models. As shown in comparative studies [56], isotropic models tend to provide reliable global deformation patterns, which were the primary focus of this parametric study, but may overestimate radial stresses while underestimating circumferential stresses carried by fibers. Our study does not aim to propose a definitive material model for embryonic valve tissues, but rather to investigate the mechanical effects of leaflet properties and anatomy. By using a consistent hyperelastic model across all simulations when altering anatomy, the relative changes in stress and leaflet deformation could be directly attributed to the parameterized anatomy, with consistent trends.

### 4.4. Biomechanical Analysis

For the biomechanical analysis of the parametric valve model developed here, a FEA approach was adopted, as it is common practice in biomechanical leaflet analysis. As noted in the literature [52,53,54], the primary advantage of FEA is its high computational efficiency, which provides an ideal framework for parametric sensitivity investigations. To quantify the mechanical burden on the leaflet tissue, the effective stress (von Mises stress) was calculated. This scalar value provides a standard measure of stress and is widely used to predict yield or failure in soft tissues. The primary trade-off of a FEA approach is the exclusion of the flow dynamics around valve leaflets (which would be instead achieved through a fully coupled FSI analysis). FEA thus sacrifices the accuracy of the dynamic interplay between the fluid and leaflets. Nevertheless, the biomechanical FEA provided here is a prerequisite for FSI methods, as it allows us to test for the structural integrity of the valve and its response to loads, prior to including the more involved interaction with the flow of blood in the model.

### 4.5. Effect of Leaflet Midline Curve on Valve Biomechanics

We performed a biomechanical analysis looking at the effect of changing the leaflet belly curve. Our analysis revealed that as the belly curve changes from a baseline curvature to a linear profile (0 to 100% shift), high-stress regions, initially distributed along the belly curve and attachment line, become progressively concentrated at the leaflet tips and the commissure edges. As in our models, numerous studies on various valve configurations have identified the commissures and attachment regions, which are regions that present sharp edges as primary sites for stress concentration [48,52,53,54,57]. The novel finding of this study that diverges from the literature is the progressive concentration of stress at the leaflet tips with increasing belly-curve shift. This dual stress localization pattern (at leaflet tips vs. commissure edges) points to a more complex biomechanical behavior than previously described.

Previous studies (on adult valve leaflets) have confirmed that the belly curve region is a critical area for stress concentration [58]. In prosthetic valves, research has shown that targeted optimization of the leaflet surface, guided by the belly curve, can significantly reduce the peak von Mises stress and lead to a smoother, more uniform stress distribution, thereby enhancing the valve’s predicted durability [48]. An important finding of the present study is the observation of a strong, linear decrease (R^2^ = 0.9875) in average effective stress as the belly curve is progressively shifted from a curved profile towards a linear shape. This result demonstrates that belly curve modifications are a highly effective and predictable means of modulating leaflet stress that could play a key role during embryonic development.

An important point for consideration is that a distinct deformation mode emerges at peak load, absent during the initial opening phase. This mode manifests as a progressive linear bending of up to 90° in the distal leaflet region as the belly curve shift increases from 0% to 100%. While this behavior is distinct from the high frequency “fluttering” described by De Hart et al. (2003) [57] or the complex “twisting” motion observed by Li and Sun (2017) [53], it is similar to the “folding” described by Smuts et al. (2011) [52] and identified as a “Shape Fold” deformation in recent durability studies [59,60], which associate it with mechanical damage. This sharp fold creates a localized ‘plastic hinge’ effect at the free edge. Computational evidence suggests that such geometric folding can drastically reduce fatigue life by up to three orders of magnitude (from >400 million to <450,000 cycles) by subjecting the collagen matrix to bending stresses that exceed its endurance limit [61]. Our results confirm that the concentration of maximum stress at the leaflet tip, which intensifies with increasing belly curve linearity, corresponds to the bending behavior observed in this very region. This bending behavior may represent a precursor mechanism leading to sliding and inappropriate coaptation [62], as well as non-calcific structural degeneration driven by mechanical stress as described in recent reviews [63]. Our study demonstrated that the belly-curve shift initiates a “folding-like” bending at the leaflet tips, which in turn may trigger a failure pathway.

While an almost linear belly curve profile yields the lowest average stress, the physiological and functional viability of such valve leaflet anatomy is a critical consideration. A belly curve with too little curvature can be detrimental, as it will limit the leaflets’ ability to displace outward, resulting in a non-optimal GOA [48] and hindering coaptation. Moreover, studies have found that actively increasing the belly curve can paradoxically decrease the GOA [49]. This finding is supported by a direct comparison in which replacing an arc-shaped radial curve with a straight-line curve was shown to increase the average GOA [62]. Our results show that the GOA exhibits a contrasting, nonlinear relationship. Interestingly, the GOA did not increase uniformly with the belly-curve change to linearity; instead, it peaked at the 70% shift and then decreased sharply at 80% (see Figure 11). Notably, the GOA at the 100% shift was not maximal and remained at a sub-optimal level, indicating that this geometry is also hemodynamically less favorable. While the 100% shift geometry (linear belly-curve profile) reduces overall leaflet stress, it is not only unrealistic but may also be functionally suboptimal during coaptation, as the shift shortens the leaflet length at the critical mid-curve. These findings suggest that, although increasing the linearity of the belly curve reduces stress, the optimal low-stress configuration at 100% shift does not necessarily represent the functionally optimal valve design. A truly optimal configuration must balance mechanical durability, hemodynamic performance (GOA), and effective leaflet sealing.

Our biomechanical aortic valve study showed an intricate interplay of leaflet anatomy and material properties. Anatomy and leaflet properties play essential roles in valve function that the embryo needs to balance as its valves are forming.

## 5. Concluding Remarks

To gain insights into aortic valve leaflet biomechanics during fetal developmental stages and assess developmental biomechanical trade-offs, this study employed a parametric leaflet model based on HH40 chick aortic valve measurements, together with in silico methodologies. Distinct assumptions were needed to overcome several limitations related to the lack of data on HH40 leaflet biomechanical properties. Our study investigated the systolic biomechanics of the developing chick embryo aortic valve, focusing on the distinct roles of leaflet material properties and belly-curve geometry. The parametric framework developed, despite its limitations, provides a robust methodology for future studies exploring functional trade-offs, particularly in the setting of valve development and congenital heart defects that are frequently accompanied by valve malformations.

The leaflet belly curve geometry emerged as a critical modulator of both mechanical stress and hemodynamic efficacy, with complex trade-offs that are balanced during leaflet development. While progressively linearizing the belly curve (0% to 100% shift) decreases average leaflet effective stress, this structural benefit is at the expense of leaflet structural damage and/or reduced hemodynamic efficiency. Indeed, the 100% shift (linear belly curve profile) while exhibiting the lowest overall stress, resulted both in a sub-optimal GOA, and induced a severe, 90° “folding-like” bending at the leaflet tips that resembled “shape-folding” modes described in durability literature [62,63] and linked to mechanical leaflet damage. Thus, our studies showed that the linear belly-curve profile induces a leaflet anatomical instability, trading global stress reduction for a local failure mode. Therefore, viable valve leaflets must develop within a functional compromise, balancing the competing demands of mechanical durability (low stress, no shape-folding), hemodynamic performance (maximizing GOA), and effective sealing via coaptation (not investigated here).

While this study offers important insights into fundamental aspects of developing valve leaflet biomechanics, it also opens new avenues for future research. The biomechanical analysis presented here could be used in the future as a basis for more involved, fully coupled FSI models of valve development that will fully incorporate the interaction between the leaflets and blood flow. Future work should also aim to create more precise and realistic 3D models of both the leaflets and the dynamics of the aortic root using high-resolution micro-CT and echocardiography imaging, partially overcoming geometric limitations encountered in the present study. This high anatomical fidelity should be complemented by experimental measurements of mechanical properties for the aortic tissue and leaflets, thus moving beyond the leaflet stiffness assumptions made in the current model. Ultimately, through an FSI model, it will be possible to accurately simulate critical diastolic events, such as valve coaptation, and analyze the effect of flow-induced systolic wall shear stress, variations in fluid pressure, and flow vortex ring formation on the biomechanics of valve leaflets both under normal and altered conditions mimicking cardiac malformations. Such a model will build upon the fundamental mechanical understanding gained in this parametric study to offer a more comprehensive and physiologically holistic perspective on developing valve biomechanics.

## Figures and Tables

**Table 2:** Mass-proportional damping coefficients (α) calculated for mode 1 of the aortic valve based on different percentages of critical damping using a natural frequency of 4779.47 rad/s. Bold values (20% critical damping) were chosen here for the HH40 aortic valve leaflets and subsequent analysis.

% of ξ = 1	α (s^−1^)
0.1	9.5589
0.5	47.7947
1	95.5894
2.5	238.9735
10	955.8940
**20**	**1911.7880**

**Table 3:** Displacement and effective stress contour plots of the eleven belly-curve-shifted valve geometries (0–100%), along with valve configurations at the ascending transition and at peak pressure. Displacement and stress legends were fixed to 0–0.3 mm and 0–0.015 MPa, respectively, for consistent comparison across all cases.

	Displacement Mag. (mm)	Effective Stress (MPa)	Ascending Transition	Peak Pressure
	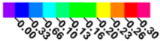	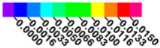	0.031 s	0.08 s
0% SHIFT	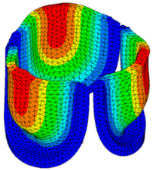	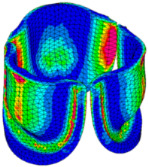	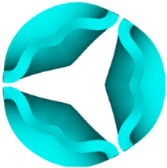	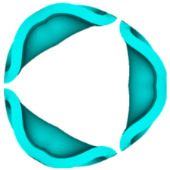
10% SHIFT	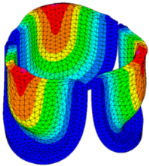	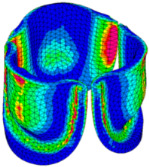	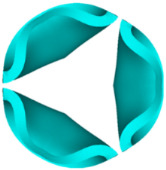	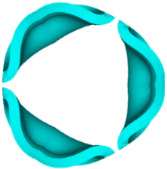
20% SHIFT	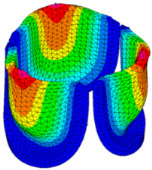	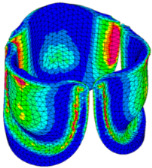	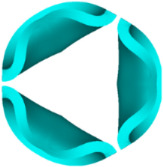	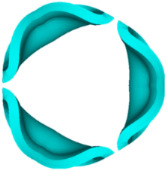
30% SHIFT	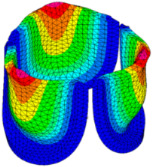	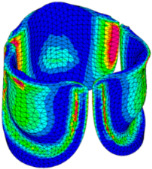	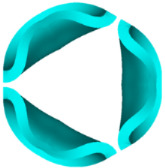	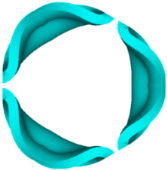
40% SHIFT	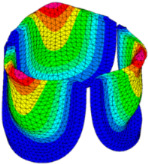	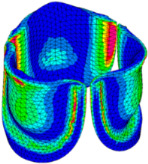	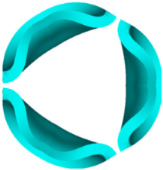	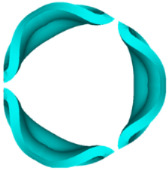
50% SHIFT	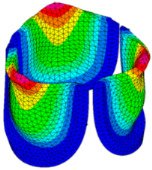	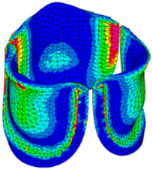	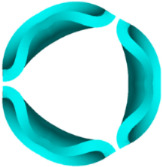	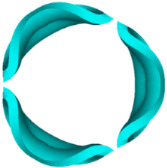
60% SHIFT	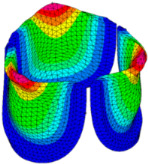	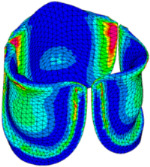	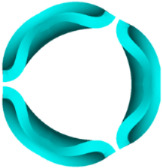	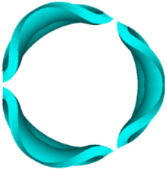
70% SHIFT	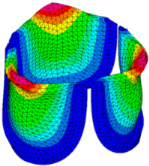	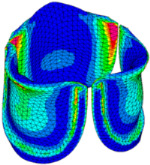	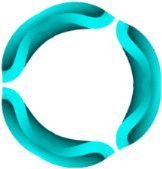	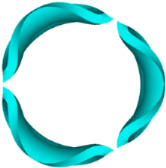
80% SHIFT	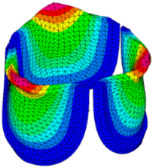	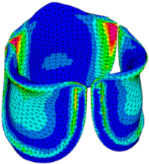	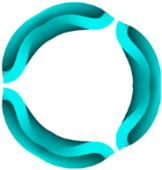	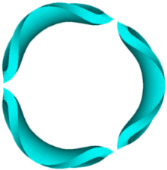
90% SHIFT	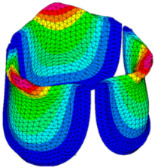	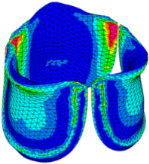	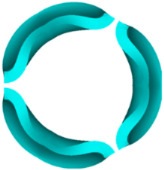	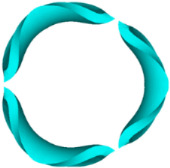
100% SHIFT	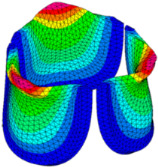	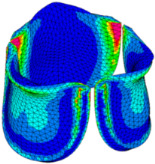	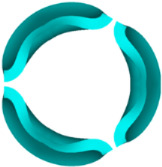	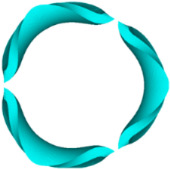

## Data Availability

The original contributions presented in this study are included in the article. Further inquiries can be directed to the corresponding author.
